# The tumor volume after radical prostatectomy and its clinical impact on the prognosis of patients with localized prostate cancer

**DOI:** 10.1038/s41598-022-09431-2

**Published:** 2022-04-09

**Authors:** Hyeong Dong Yuk, Seok-Soo Byun, Sung Kyu Hong, Hakmin Lee

**Affiliations:** 1 Department of Urology, Seoul National University Hospital, 101 Daehak-ro, Jongno-gu, Seoul, 110-744 Korea; 2 Department of Urology, Seoul National University Bundang Hospital, 82, Gumi-ro 173 Beon-gil, Bundang-gu, Seongnam-si, Gyeonggi-do 13620 Korea

**Keywords:** Medical research, Urology

## Abstract

We evaluated the contribution of tumor volume (TV) to localized prostate cancer (PCa) patients’ prognosis. We retrospectively analyzed the data of 2394 patients who underwent radical prostatectomy (RP) for localized PCa. The effect of TV and tumor prostate ratio (TV/PV) on PCa patients' prognosis was analyzed through Kaplan–Meier and Cox-proportional analysis. The mean prostate volume for all patients was 36.5 ± 15.4 cc, and the mean TV was 5.9 ± 8.3 cc. A significant positive relationship was observed between the classification by risk group in D’ Amico risk classification and the National Comprehensive Cancer Network risk group (P < 0.001). The high TV showed significantly worse pathologic outcomes than the low TV in terms of high rates of extra-capsular extension, seminal vesicle invasion, and positive surgical margin (P < 0.05). The patients with high TV and TV/PV had significantly shorter biochemical recurrence-free survivals than those with low TV and TV/PV (P < 0.001). Finally, based on multivariate Cox-proportional analyses, TV and TV/PV was an independent predictor to predict shorter biochemical recurrence-free survival as both a TV (HR: 1.04, 95% CI 1.04–1.05, P < 0.001) and TV/PV (HR: 1.42, 95% CI 1.13–1.78, P = 0.003). TV was revealed to be an independent prognostic factor in the postoperative biochemical recurrence. Patients with a high number of positive core and longer tumor length were significantly related to higher TV.

## Introduction

Prostate cancer (PCa) is the most frequently diagnosed malignancy and fifth major cause of cancer-related deaths in the United States of America^1^. PCa has unique characteristics, including multi-focality in number and heterogeneity in size and grade^2, 3^. Tumor volume (TV) is considered a significant prognostic factor for biochemical recurrence (BCR)-free survival, positive surgical margin, and overall survival in patients with PCa^2, 4–12^. Other studies produced conflicting results that TV has no prognostic value when combined with pathologic stage or Gleason score^13–17^. Most recently, Uhlman et al. analyzed TV and tumor percentage involvement in a large cohort of 3528 participants and concluded that TV did not show significant results in the multivariate analysis but tumor percentage involvement was predictive of BCR after radical prostatectomy^18^. However, exact imaging or estimating tools to predict the TV of each patient preoperatively are unavailable. Even though the multiparametric magnetic resonance imaging (MRI) has excellent ability to delineate the shape and size of clinically significant PCa, many patients have MRI-invisible clinically-confirmed PCa^19^.

Focal therapy is now gaining more attention because of its ability to partially treat PCa and preserve erectile function and urinary continence. Numerous energy sources have been developed for the focal ablation of PCa, but the weak aspect of focal therapy is that precise tumor recognition is difficult and is impossible when conventional imaging modalities are used. However, it would be quite helpful from the standpoint of performing focal therapy to select the best candidate, if we can understand and predict TV in PCa patients. Therefore, we tried to evaluate the actual contribution of TV in localized PCa patients, analyze the prognostic value of TV, and find the significant predictor for large TVs preoperatively.

## Methods

The present study does not contain clinical studies or patient data and permitted by Institutional Review Board (IRB No. B-1906-549-101) of Bundang Seoul National University Bundang Hospital. The patient's written informed consent was waived for a retrospective analysis of the prospective database. Study protocol and contents associated with this study followed the Declaration of Helsinki guidelines. After the approval of the institutional review board, we retrospectively analyzed the data of 2394 patients who underwent radical prostatectomy (RP) for localized PCa from May 2006 to January 2017 at our institution. After further exclusion of some patients (preoperative radiation therapy [n = 4], adjuvant or neoadjuvant hormone therapy [n = 43], and incomplete information [n = 31]), we finally analyzed a total of 2316 patients. Clinical and pathological information were retrieved from our institutional database, which is prospectively maintained. The RP was performed by open retro-pubic, laparoscopic, or robot-assisted approach. The TNM staging system was used to evaluate tumors according to the 6th edition of the American Joint Committee Cancer guidelines, and adverse pathologic outcomes, including seminal vesicle invasion (SVI), extraprostatic extension (EPE), and positive surgical margin (PSM), were evaluated as previously described. All pathologic evaluations were performed using the modified definition of the 2005 International Society of Urological Pathology Consensus Conference. TV was assessed as described below. After the fixation of pathologic specimens using 20% buffered formalin, the surgical specimens were sectioned in 5 mm intervals. The pathologist determined the number of tumors and dimensions of tumor area from every axial slice of each specimen. Subsequently, TV was calculated by multiplying the percentage of tumor area to the total prostate volume^20, 21^. The tumor prostate ratio (TV/PV) was calculated as the ratio of the tumor volume to the prostate volume. Postoperative follow-ups were usually performed in 2 or 3 month intervals for the initial 2 years and annually thereafter, in case of no evidence for biochemical recurrence (BCR). The BCR was defined as the continuous elevation of prostate-specific antigen (PSA) over 0.2 ng/ml in more than two consecutive tests.

The independent t-tests, Wilcoxon rank sum test, Chi-square test and Fisher’s exact test were performed to compare the clinical or pathologic variables between subgroups. Multiple regression tests were performed to identify the possible relationship among the variables; Kaplan–Meier and Cox proportional hazard analyses also compared the survival outcomes among the groups. All statistical analyses were performed using the SPSS software (SPSS 19.0, Chicago, IL, USA); all P values were presented as two-sided; P values < 0.05 were considered to be statistically significant.

## Results

**Figure 1:**
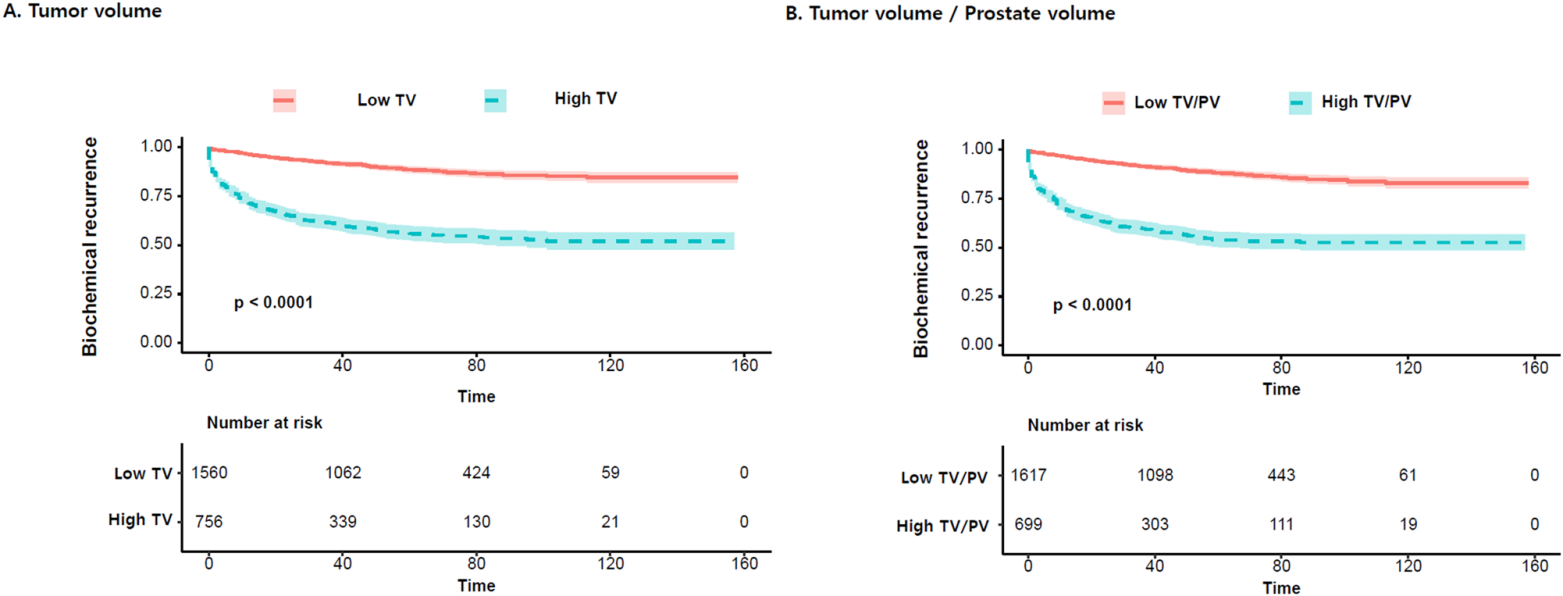
Kaplan–Meier analysis on the biochemical recurrence free survival according to the tumor volume.

**Figure 2:**
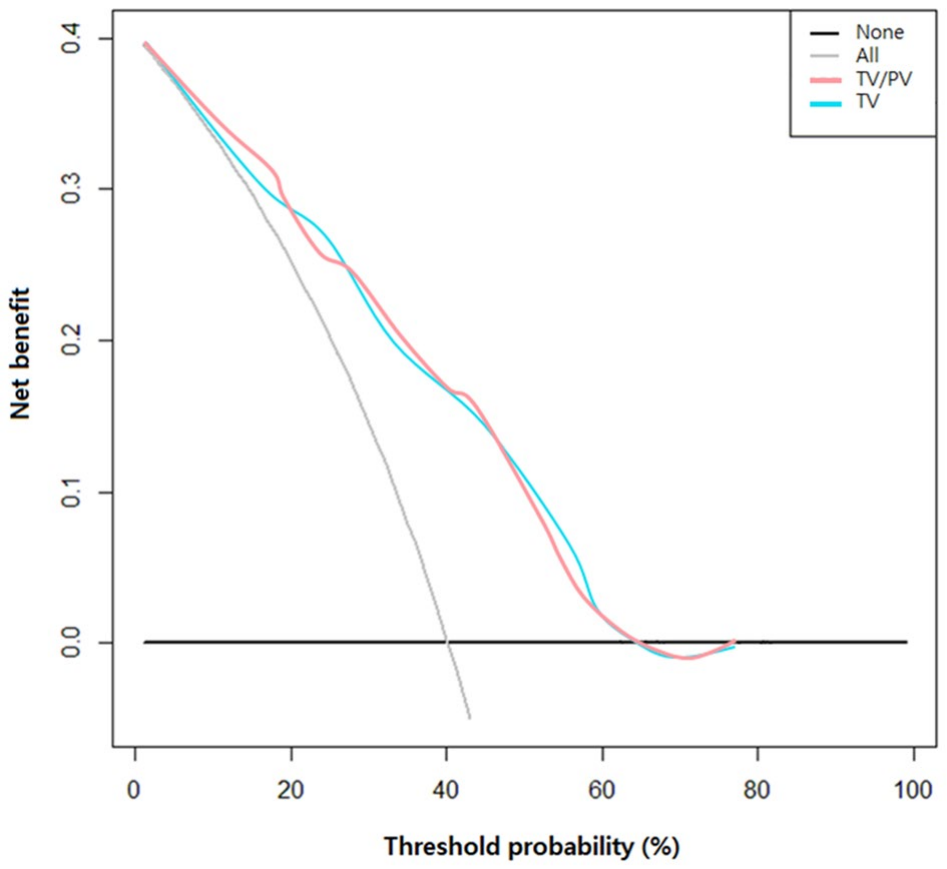
Decision curve analysis of TV and TV/PV for biochemical recurrence evaluated.

Clinical characteristics and pathologic outcomes were summarized in Table 1. The mean prostate volume for all patients was 36.5 ± 15.4 cc and mean TV was 5.9 ± 8.3 cc (Table 1). We observed significant positive relationship between increased risk groups and TV and TV/PV (P < 0.001). After stratification of patients by the D’Amico risk classification system, the low risk, intermediate risk, and high risk groups had a mean TV of 2.7 ± 3.2 cc, 4.2 ± 4.0 cc, and 11.6 ± 12.5 cc, respectively (Table 2). When we subsequently subdivided the patients according to the National Comprehensive Cancer Network (NCCN) risk groups, TV was 2.0 ± 3.5 cc for the very low risk group, 3.1 ± 3.3 cc for the low risk group, 3.2 ± 3.7 cc for the favorable intermediate risk group, 5.2 ± 4.8 cc for the unfavorable intermediate risk group, 11.1 ± 12.0 cc for the high risk group, and 20.0 ± 16.2 cc for the very high risk group. TV/PV was 0% for the very low risk group, 10% for the low risk group, 10% for the favorable intermediate risk group, 10% for the unfavorable intermediate risk group, 20% for the high risk group, and 50% for the very high risk group. We observed a significant positive relationship between the ranks of the NCCN risk groups and TV and TR (P < 0.001). However, no significant difference existed between prostate volume, according to the D’Amico and NCCN risk group classification (P > 0.05). Analysis of the receiver operating curve of TV to have biochemical recurrence revealed that TV of 5.27 cc indicated the maximal Youden’s score, whereas area under the curve of TV was revealed as 0.778 [95% confidence interval (CI) 0.755–0.801]. And Analysis of the receiver operating curve of TV/PV to have biochemical recurrence revealed that TV/PV of 16.7% indicated the maximal Youden’s score, whereas area under the curve of TV/PV was revealed as 0.781 [95% confidence interval (CI) 0.759–0.804]. Subsequently, patients were grouped into high and low TV and TV/PV by using the cut-off of 5.27 cc and 16.7%. All the participants were grouped into high TV and low TV, and high TR and low TR (Table 1). The high TV and TV/PV indicated significantly worse clinical characteristics, in terms of high PSA (P < 0.001); pathologic stage (P < 0.001); and rate of EPE, SVI, (Table 1). Multivariate multiple regression tests revealed that preoperative PSA, clinical stage, D’Amico risk group, ratio of positive biopsy cores (positive/total), and tumor length (longest among the positive biopsies) showed significant relationship to high TV and high TV/PV (Table 3). The Kaplan–Meier analysis showed that the high TV and TV/PV group showed significantly shorter biochemical recurrence-free survival than the low TV and TV/PV (P < 0.001) (Fig. 1). The Clninical value of TV and TV/PV was similar (Fig. 2). Table 4 shows the Cox proportional hazard analysis of significant predictors and covariates for biochemical recurrence in high tumor volume prostate cancer. Finally, the multi-variate Cox-proportional model revealed that TV and TV/PV was an independent predictor to predict shorter biochemical recurrence-free survival as both a TV (HR: 1.01, 95% CI 1.00–1.02, P < 0.001) and TV/PV (HR: 1.00, 95% CI 0.99–1.01, P = 0.003).

**Table 1:** The clinical characteristics and pathologic outcomes according to tumor volume and tumor prostate ratio.

Median (interquartile range) or number (percent)	Total (N = 2316)	Tumor volume		P value	Tumor prostate ratio		P value
		Low (N = 1560)	High (N = 756)		Low (N = 1294)	High (N = 1022)	
Age (year)	67.0 (62.0–71.0)	67.0 (61.0–71.0)	68.0 (63.0–72.0)	0.001**	67.0 (61.0–71.0)	67.0 (62.0–71.0)	0.553**
Body mass index (kg/m^2^)	24.4 ± 2.8	24.3 ± 2.7	24.5 ± 3.0	0.011**	24.4 ± 2.7	24.4 ± 2.9	0.481**
**Damico risk classification**				< 0.001^†^			< 0.001^†^
Low	699 (30.2%)	626 (40.1%)	73 (9.7%)		549 (42.4%)	150 (14.7%)	
Intermediate	936 (40.4%)	684 (43.8%)	252 (33.3%)		553 (42.7%)	383 (37.5%)	
High	681 (29.4%)	250 (16.0%)	431 (57.0%)		192 (14.8%)	489 (47.8%)	
**NCCN risk classification**				< 0.001^†^			< 0.001^†^
Very low	150 (6.9%)	139 (9.7%)	11 (1.5%)		134 (11.3%)	16 (1.6%)	
Low	423 (19.4%)	371 (25.8%)	52 (7.0%)		310 (26.2%)	113 (11.4%)	
Favorable intermediate	416 (19.1%)	356 (24.8%)	60 (8.1%)		309 (26.1%)	107 (10.8%)	
Unfavorable intermediate	523 (24.0%)	327 (22.8%)	196 (26.5%)		243 (20.5%)	280 (28.2%)	
High	626 (28.8%)	236 (16.4%)	390 (52.7%)		181 (15.3%)	445 (44.9%)	
Very high	39 (1.8%)	8 (0.6%)	31 (4.2%)		8 (0.7%)	31 (3.1%)	
Prostate specific antigen (ng/mL)	13.0 ± 16.5	8.5 ± 9.2	22.4 ± 23.1	< 0.001**	8.2 ± 9.2	19.1 ± 21.1	< 0.001**
Prostate volume (g)	36.6 ± 15.4	36.1 ± 14.7	37.4 ± 16.8	0.103**	38.6 ± 15.7	34.0 ± 14.7	< 0.001**
**Clinical T stage**				< 0.001^†^			< 0.001^†^
T1	1415 (61.1%)	1086 (69.6%)	329 (43.5%)		925 (71.5%)	490 (47.9%)	
T2	673 (29.1%)	421 (27.0%)	252 (33.3%)		332 (25.7%)	341 (33.4%)	
≥ T3	228 (9.8%)	53 (3.4%)	175 (23.1%)		37 (2.9%)	191 (18.7%)	
**Biopsy Gleason score**				< 0.001^†^			< 0.001^†^
6	921 (39.8%)	769 (49.3%)	152 (20.1%)		675 (52.2%)	246 (24.1%)	
7	982 (42.4%)	625 (40.1%)	357 (47.2%)		489 (37.8%)	493 (48.2%)	
≥ 8	413 (17.8%)	166 (10.6%)	247 (32.7%)		130 (10.0%)	283 (27.7%)	
**Pathologic results on biopsy**							
Number of positive cores	3.8 ± 2.8	2.8 ± 2.0	5.7 ± 3.3	< 0.001**	2.5 ± 1.8	5.3 ± 3.1	< 0.001**
Percentage of positive cores (%)	31.8 ± 23.0	23.6 ± 16.4	49.8 ± 25.2	< 0.001**	21.0 ± 14.2	46.3 ± 24.5	< 0.001**
Median maximal tumor length (cm)	0.5 (0.3–0.8)	0.4 (0.2–0.6)	0.9 (0.5–1.3)	< 0.001**	0.3 (0.2–0.6)	0.8 (0.5–1.2)	< 0.001**
Median length of core (cm)	1.7 (1.4–1.9)	1.7 (1.4–1.8)	1.7 (1.4–1.9)	0.037**	1.7 (1.4–1.8)	1.7 (1.4–1.9)	0.104**
Maximal tumor involvement of positive core (%)	38.0 ± 31.1	28.6 ± 22.0	57.5 ± 37.4	< 0.001**	26.4 ± 21.3	53.0 ± 35.0	< 0.001**
Prostate volume (g)	36.0 (30.0–46.0)	36.0 (30.0–44.0)	38.7 (32.0–48.0)	< 0.001**	38.0 (32.0–48.0)	35.0 (30.0–43.0)	< 0.001**
Tumor volume (g)	3.3 (1.6–6.6)	2.2 (1.2–3.4)	9.3 (6.8–15.1)	< 0.001**	1.8 (1.0–2.8)	7.2 (4.9–12.0)	< 0.001**
Tumor prostate ratio (%)					0.1 ± 0.0	0.3 ± 0.2	< 0.001**
**Pathologic T stage**				< 0.001^†^			< 0.001^†^
T2b	221 (9.5%)	209 (13.4%)	12 (1.6%)		204 (15.8%)	17 (1.7%)	
T2c	1354 (58.5%)	1099 (70.4%)	255 (33.7%)		931 (71.9%)	423 (41.4%)	
T3a	505 (21.8%)	209 (13.4%)	296(39.2%)		132(10.2%)	373(36.5%)	
≥ T3b	236 (10.2%)	43 (2.8%)	193 (25.5%)		27 (2.1%)	209 (20.5%)	
Pathologic N1 stage	59 (4.4%)	6 (0.9%)	53 (8.3%)	< 0.001^†^	6 (1.1%)	53 (6.6%)	< 0.001^†^
**Pathologic Gleason score**				< 0.001^†^			< 0.001^†^
6	168 (7.3%)	157 (10.1%)	11 (1.5%)		156 (12.1%)	12 (1.2%)	
7	1824 (78.8%)	1296 (83.1%)	528 (69.8%)		1051 (81.2%)	773 (75.6%)	
8	118 (5.1%)	63 (4.0%)	55 (7.3%)		53 (4.1%)	65 (6.4%)	
≥ 9	206 (8.9%)	44 (2.8%)	162 (21.4%)		34 (2.6%)	172 (16.8%)	
Multifocality (yes)	1884 (81.3%)	1309 (83.9%)	575 (76.1%)	< 0.001^†^	1078 (83.3%)	806 (78.9%)	0.006^†^
Extracapsular extension	714 (30.8%)	237 (15.2%)	477 (63.1%)	< 0.001^†^	145 (11.2%)	569 (55.7%)	< 0.001^†^
Seminal vesicle invasion	231 (10.0%)	46 (2.9%)	185 (24.5%)	< 0.001^†^	28 (2.2%)	203 (19.9%)	< 0.001^†^

*ECE* extracapsular extension, *GS* Gleason-score, *NCCN* National Comprehensive Cancer Network, *PSA* prostate specific antigen, *SVI* seminal vesical invasion.

*Independent t-test.

**Wilcoxon rank sum test.

^†^Chi-square test.

^††^Fisher's exact test.

**Table 2:** Mean tumor volume difference according to NCCN and D’Amico risk classification.

D’Amico risk classification	Low (N = 699)	Intermediate (N = 936)	High (N = 681)	p-value			
Median prostate volume	36.0 (30.0–46.0)	36.0 (30.0–44.0)	38.0 (32.0–48.0)	< 0.001**			
Median tumor volume	1.9 (0.9–3.4)	3.1 (1.7–5.6)	7.2 (3.6–14.8)	< 0.001**			
Median tumor prostate ratio	0.1 (0.0–0.1)	0.1 (0.1–0.2)	0.2 (0.1–0.4)	< 0.001**			
Multifocality (yes)	591 (84.5%)	788 (84.2%)	505 (74.2%)	< 0.001^†^			

NCCN risk classification	Very low (N = 160)	Low (N = 450)	Favorable intermediate (N = 443)	Unfavorable intermediate (N = 556)	High (N = 666)	Very high (N = 41)	P value
Median prostate volume	44.0 (36.0–55.0)	34.0 (29.0–44.0)	37.0 (30.0–46.0)	35.0 (30.0–44.0)	39.0 (32.0–48.0)	36.0 (30.0–50.0)	< 0.001**
Median tumor volume	1.1 (0.6–2.1)	2.3 (1.2–4.0)	2.4 (1.3–3.8)	4.1 (2.3–6.6)	7.2 (3.5–14.0)	15.0 (7.2–34.8)	< 0.001**
Median tumor prostate ratio	0.0 (0.0–0.0)	0.1 (0.0–0.1)	0.1 (0.0–0.1)	0.1 (0.1–0.2)	0.2 (0.1–0.4)	0.5 (0.2–0.7)	< 0.001**
Multifocality (yes)	122 (81.3%)	358 (84.6%)	364 (87.5%)	430 (82.2%)	465 (74.3%)	25 (64.1%)	< 0.001^†^

*NCCN* National Comprehensive Cancer Network, *RP* radical prostatectomy.

*Independent t-test.

**Wilcoxon rank sum test.

^†^Chi-square test.

^††^Fisher's exact test.

**Table 3:** Multivariate regression analysis for high tumor volume and high tumor volume ratio prostate cancer predictor.

Variable	Tumor volume		Tumor prostate ratio	
	OR (95% CI)	P value	OR (95% CI)	P value
Age	1.02 (1.00–1.04)	0.073	0.99 (0.98–1.01)	0.430
Body mass index	1.02 (0.97–1.06)	0.472	1.00 (0.97–1.04)	0.834
Diabetes mellitus (ref = 0)	1.00 (0.76–1.32)	0.980	1.13 (0.88–1.46)	0.334
Prostate specific antigen	1.07 (1.05–1.09)	< 0.001	1.08 (1.06–1.10)	< 0.001
**D’Amico risk classification**		0.030		0.035
Low	Reference		Reference	
Intermediate	1.64 (1.04–2.59)	0.033	1.06 (0.69–1.63)	0.030
High	2.01 (1.07–3.79)	0.030	1.46 (1.02–1.98)	0.035
Clinical stage ≥ T3	2.41 (1.47–3.93)	< 0.001	2.64 (1.52–4.57)	0.001
Prostate volume	1.01 (1.00–1.02)	0.003	0.97 (0.96–0.98)	< 0.001
**Biopsy Gleason score**		0.547		0.811
Low	Reference		Reference	
Intermediate	0.83 (0.57–1.22)	0.347	1.12 (0.76–1.65)	0.555
High	0.76 (0.44–1.31)	0.329	1.01 (0.56–1.84)	0.962
Number of positive cores	1.30 (1.23–1.37)	< 0.001	1.36 (1.29–1.44)	< 0.001
Maximal tumor involvement of positive core	1.02 (1.01–1.02)	< 0.001	1.01 (1.01–1.02)	0.001
Median maximal tumor length	1.80 (1.07–3.01)	0.027	1.75 (1.00–3.04)	0.050

**Table 4:** Cox proportional hazards analyses of significant predictors, covariates on biochemical recurrence.

	Tumour volume high^a)^													
	LNI						Stage T3							
	Tumour volume high	P value	Stage T3	P value	Positive surgical margin	P value	Positive surgical margin	P value	Gleason: low	P value	Gleason: intermediate	P value	Gleason: high	P value
**Covariates**														
PSA (units = 10%)	1.01 (1.01–1.01)	< 0.001	1.00 (0.99–1.01)	0.781	1.00 (0.99–1.01)	0.947	1.01 (1.00–1.01)	< 0.001	–	–	1.02 (1.01–1.02)	< 0.001	1.00 (1.00–1.01)	0.087
Age (years)	1.00 (0.99–1.02)	0.531	0.98 (0.95–1.02)	0.436	1.00 (0.96–1.05)	0.884	1.01 (0.99–1.02)	0.568	–	–	1.00 (0.98–1.02)	0.887	1.00 (0.98–1.03)	0.967
**Significant predictors**														
Pathological Gleason score (sum)	1.72 (1.56–1.89)	< 0.001	1.28 (0.93–1.77)	0.132	1.49 (1.01–2.18)	0.044	1.71 (1.50–1.95)	< 0.001						
Positive surgical margin (Ref: 0)	1.52 (1.29–1.79)	< 0.001	1.40 (0.66–2.98)	0.375					–	–	1.25 (0.98–1.59)	0.068	1.85 (1.32–2.61)	< 0.001
**Pathological stage**														
Stage T3 (Ref: stage T2)	2.94 (2.47–3.51)	< 0.001			–									
**Lymph node invasion**														
Yes (Ref: No)	2.36 (1.74–3.19)	< 0.001					2.37 (1.67–3.37)	< 0.001	–	–	3.18 (1.92–5.27)	< 0.001	2.40 (1.61–3.59)	< 0.001
LN Not removed (Ref: removed)	0.43 (0.37–0.51)	< 0.001												

	Tumour percentage high^b)^													
	Positive surgical margin	P value	Stage T2	P value	LNI	P value	Stage T3	P value	Gleason: low	P value	Gleason: intermediate	P value	Gleason: high	P value
**Covariates**														
PSA (units = 10%)	1.01 (1.00–1.01)	< 0.001	1.02 (1.02–1.02)	< 0.001	1.00 (0.99–1.01)	0.732	1.01 (1.01–1.01)	< 0.001	1.03 (0.88–1.21)	0.686	1.02 (1.01–1.02)	< 0.001	1.00 (1.00–1.01)	0.031
Age (years)	1.00 (0.99–1.02)	0.721	1.00 (0.98–1.02)	0.740	0.98 (0.94–1.02)	0.312	0.99 (0.98–1.01)	0.478	0.97 (0.86–1.10)	0.646	1.00 (0.98–1.01)	0.805	0.99 (0.97–1.01)	0.377
**Significant predictors**														
Pathological Gleason score (sum)	1.75 (1.54–1.99)	< 0.001	1.99 (1.60–2.46)	< 0.001	1.35 (0.97–1.87)	0.077	1.72 (1.53–1.92)	< 0.001						
Positive surgical margin (Ref: 0)			2.28 (1.70–3.06)	< 0.001	1.36 (0.64–2.89)	0.429	1.46 (1.20–1.79)	< 0.001	25.67 (3.87–170.42)	0.001	1.49 (1.22–1.82)	< 0.001	2.17 (1.53–3.09)	< 0.001
**Pathological stage**														
Stage T3 (Ref: stage T2)	2.73 (2.04–3.65)	< 0.001			2.14 (0.43–10.59)	0.349			0.89 (0.12–6.39)	0.909	3.47 (2.86–4.23)	< 0.001	4.80 (2.71–8.49)	< 0.001
**Lymph node invasion**														
Yes (Ref: No)	2.40 (1.68–3.42)	< 0.001	9.73 (2.74–34.56)	< 0.001			2.63 (1.91–3.63)	< 0.001	–	–	3.18 (1.93–5.23)	< 0.001	2.45 (1.64–3.66)	< 0.001

*LN* not removed (Ref: removed).

## Discussion

In this study, we investigated the overall contribution of TV and TV/PV according to the risk group systems from D’Amico criteria and NCCN guidelines and observed that TV significantly increased in the higher risk groups of both systems. The patients of the low risk group exhibited a TV of 2–3 cm^3^, with a tumor diameter between 1.4 and 1.6 cm when the tumor is assumed to be spherical, and a TV between 1.3 and 1.4 cm when the tumor is assumed to be a regular hexahedron. Large tumors awere significantly related to an increased postoperative BCR in our multi-variate Cox proportional hazard analyses. The preoperative PSA, clinical stage, prostate volume, percentage of positive biopsy cores, and maximal tumor length among positive biopsy cores were significantly related to high TV and high TV/PV in our multi-variate regression tests.

TV has been presented as a significant prognostic factor of PCa in several papers^2, 4–12^. Stamey et al. reported that cancer volume and high Gleason score were significantly related with worse BCR-free survival after analyzing the results of 379 patients who were treated with radical prostatectomy^2^. They argued that the exact way to predict cancer volume may be helpful in predicting the outcomes of PCa surgery. Subsequently, Nelson et al. investigated the effect of TV on pathologic outcomes and biochemical recurrence after surgery^5^. They concluded that TV directly correlated with pathological stage and PSA recurrence after radical prostatectomy; therefore, TV was revealed as an independent predictor for worse prognosis in patients with localized PCa. Another study by Chun et al. showed that cancer volume and high Gleason score were the independent predictors for postoperative BCR in their relatively large cohort of 780 participants treated with radical prostatectomy^4^. Furthermore, another study by Uhlman et al. analyzed data from a large cohort of 3528 participants who were treated with radical prostatectomy to evaluate the prognostic influence of TV^16^. They analyzed TV with BCR-free survival, even though significant difference existed in their BCR-free survival rates after univariate Kaplan–Meier analyses.

However, there were other studies showing contradictory results regarding the use of TV as a predictive prognostic factor^13–16^. Kikuchi et al. also analyzed a larger cohort of 1302 participants who were also treated with radical prostatectomy. They observed only a weak relationship with postoperative prognosis, but no statistically significant results were obtained from multi-variate analyses^13^. Merrill et al. analyzed a large cohort of 1833 participants who were also treated with radical prostatectomy and found that TV was significantly associated with a higher rate of BCR in the high pathologic Gleason score subgroup (≥ 3 + 4), but not in the Gleason 6 subgroup (3 + 3)^15^. Another study by May et al. reported that absolute TV was unable to predict postoperative BCR, unlike relative TV (TV/total prostate volume), which showed significant results in predicting prognosis^14^. However, in our study, TV and TV/PV were significant independent predictors predicting BCR. As for the accuracy of prediction, TV/PV and TV do not have a big difference, but it is easier and more beneficial to use TV/PV than TV (Fig. 2; Supplementary Fig. 1). And in our study, high TV was associated with worse clinical characteristics such as worth pathologic state, rate of EPE, SVI, high-risk group, more positive biopsy core, and longer tumor length. There is no consensus on this, but men with larger prostate will have higher testosterone levels than men with smaller prostate. It may be associated with more aggressive prostate cancer. It can be estimated that the more aggressive prostate cancer, the more active cell-to-cell migration or progression will be.

Lately, focal therapy is gaining more attention with clinical benefits in terms of better erectile and urinary functions after treatment^22^. However, there are still limitations for focal therapy, because tumor location cannot be exactly predicted using conventional imaging modalities and biopsy protocols^23^. The understanding of the epidemiology of TV is quite important when planning and finding optimal candidates for focal therapy. We observed from our results that the mean TV was about 2.0 cc for very low risk, 3.1 cc for low risk, and 3.2 cc for favorable intermediate risk group. Patients in the unfavorable intermediate risk group showed significantly larger TV than those in the favorable intermediate risk group (3.2 ± 3.7 cc versus 5.2 ± 4.8 cc, P < 0.001). From our volumetric results of TV, we believe that the optimal candidates for focal therapy could be patients between very low risk and favorable intermediate risk groups. However, the patients in the very low risk group should be recommended for active surveillance.

Our study is certainly not without limitations. First, it is limited by its retrospective analyses, even though the data on TV was prospectively accumulated. Second, there is a possibility of selection bias since we only included patients treated with radical prostatectomy. Third, the present study only analyzed the total TV but not the detailed pathologic information about number of tumors and location, which is also important for prognosis of PCa patients. Even so, we believe that we provided the most recent data regarding the actual epidemiology of TV in patients treated with radical prostatectomy, which makes our study clinically meaningful.

## Conclusion

TV of PCa was revealed to be an independent prognostic factor in predicting postoperative biochemical recurrence, showing a significantly positive relationship with increase in risk in the risk group system. Further study with more detailed data on tumor location and multifocality is required to understand the nature of PCa.

## Supplementary Information

Supplementary Figure 1.
