# Supplementary figures and images for: The tumor volume after radical prostatectomy and its clinical impact on the prognosis of patients with localized prostate cancer
Source: Sci Rep. 2022 Apr 9;12:6003. doi: 10.1038/s41598-022-09431-2 (PMC8994775; doi:10.1038/s41598-022-09431-2)

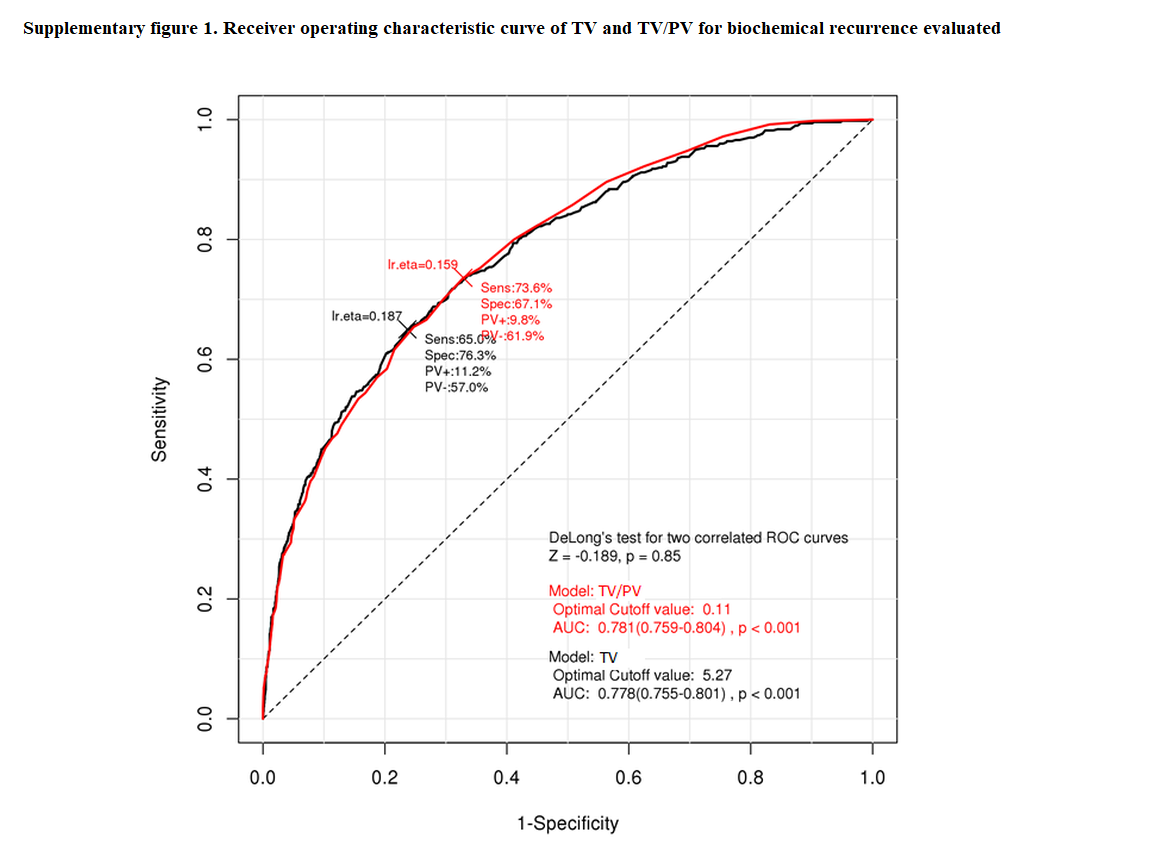

Supplement: Supplementary file 1 — Supplementary Figure 1. [file 41598_2022_9431_MOESM1_ESM.tif]
